# Predictive biomarkers of placebo response in patients with cancer: an exploratory analysis of the ABCD randomized clinical trial

**DOI:** 10.1007/s00520-026-10689-9

**Published:** 2026-04-22

**Authors:** David Hui, Kristofer Jennings, Amy Ontai, Sandra K. Hanneman, Eduardo Bruera

**Affiliations:** 1 Department of Palliative, Rehabilitation and Integrative Medicine, Unit 1414, University of Texas MD Anderson Cancer Center, 1515 Holcombe Boulevard, Houston, TX 77030 USA; 2 Center for Goal Concordant Care Research, The University of Texas MD Anderson Cancer Center, Houston, TX USA; 3 Department of General Oncology, The University of Texas MD Anderson Cancer Center, Houston, TX USA; 4 Department of Biostatistics, The University of Texas MD Anderson Cancer Center, Houston, TX USA; 5 Department of Research, Cizik School of Nursing, The University of Texas Health Science Center at Houston, Houston, TX USA

**Keywords:** Biomarkers, Placebo effect, Patient reported outcome measures, Palliative care, Neoplasms

## Abstract

**Purpose:**

Placebo effect is commonly observed in symptom management randomized clinical trials. However, there are currently no established predictive biomarkers of placebo response. We examined the patterns of association between placebo response and baseline plasma cytokine levels for 10 symptoms in patients with cancer.

**Methods:**

This is an exploratory analysis of the double-blind, randomized ABCD trial, which compared high-dose dexamethasone to placebo for dyspnea treatment in patients with cancer (clinicaltrials.gov; NCT03367156). The primary outcome was change in dyspnea intensity. Four plasma cytokines (TNF, IL-6, IL-8, and IL-10) and 10 Edmonton Symptom Assessment System symptoms were measured at baseline, day 7, and day 14. Generalized additive models and exploratory analyses were used to examine the patterns of association between baseline cytokine levels and symptom responses on day 7 and day 14 in placebo and dexamethasone groups.

**Results:**

This analysis included data from 45 patients. In the placebo group, we observed a significant negative association (*p* < 0.05) between ≥ 1 of the measured cytokines’ baseline levels across multiple symptoms on day 7 (5/10 symptoms) and day 14 (3/10 symptoms), including appetite, drowsiness, nausea, and pain. Exploratory analysis revealed a negative association in the placebo group in 33/40 cytokine-symptom response combinations for day 7 (*p *< 0.001) and 29/40 combinations for day 14 (*p* = 0.006).

**Conclusion:**

Our preliminary findings suggest patients with high baseline cytokine levels may be less likely to report placebo response for multiple symptoms. Upon further confirmation, these findings may have implications for future clinical trial design.

**Structured abstract:**

In this exploratory analysis, we observed that patients with cancer were less likely to report placebo response for multiple symptoms if they had higher levels of baseline cytokines. These preliminary findings have important potential implications for placebo-controlled clinical trials, including patient selection, design of interventional controls, and trial interpretation. Additionally, they invite further research on possible mechanistic links between inflammation and placebo effect.

## Introduction

Placebo response, or any observed improvement in the placebo group, has been commonly reported in supportive care clinical trials examining pharmacologic therapies to improve patient-reported outcomes [1–4]. In these trials, multiple symptoms have been found to significantly respond to placebo, including dyspnea, fatigue, pain, and anorexia and cachexia [1–4]. The presence of placebo response often confounds interpretation of these studies, as it may reduce the observed effect size of the intervention or result in negative trials. Beyond this basic characterization, little is known about the mechanism of placebo response across multiple symptoms, specifically in the cancer palliative care setting.

This observation highlights the need to better understand the placebo response and its predictors, with implications for patient selection, design of the active and control interventions, and trial interpretation. However, studies on placebo response biomarkers are nascent, with a scarcity of such research in the cancer palliative care setting. A handful of randomized clinical trials in depression, gastrointestinal disorders, and pneumonia suggest that baseline levels of inflammatory cytokines may be associated with placebo response, but these studies had heterogenous outcomes and definitions of response, as well as mixed findings on clinical significance [5–10]. Thus, further research is needed to examine cytokines as predictive biomarkers for placebo response.

In a recent pre-planned secondary analysis of the Alleviating Breathlessness in Cancer Patients with Dexamethasone (ABCD) trial, we examined the predictive utility of baseline cytokine levels for dyspnea response to dexamethasone or placebo in patients with cancer [11, 12]. We found that higher baseline cytokine levels were associated with a greater reduction in dyspnea for dexamethasone as well as a lower reduction in dyspnea for placebo. However, this study only examined one symptom, and we hypothesize that placebo response for multiple symptoms may be associated with baseline cytokines. In this current exploratory analysis, we examined the patterns of association between placebo response and baseline cytokine levels for all 10 Edmonton Symptom Assessment System (ESAS) symptoms in patients with cancer.

## Materials and methods

### Study design

This is a secondary, exploratory analysis of the double-blind, placebo-controlled, parallel group ABCD trial (clinicaltrials.gov; NCT03367156) to examine the patterns of association between symptom response to placebo or dexamethasone and baseline cytokines in patients with cancer. The ABCD trial has been described previously [11, 12]. Briefly, patients were randomized in a 2:1 ratio to receive 8 mg oral dexamethasone every 12 h for 7 days and then 4 mg every 12 h for another 7 days or a matching placebo. The primary outcome was patient-reported dyspnea levels, which were assessed by an 11-point numeric rating scale (NRS) [13]. Research staff conducting study assessments, attending physicians, and patients were masked to allocation of study medications and outcomes. Only the study pharmacist had access to allocation data. The Institutional Review Board of the University of Texas MD Anderson Cancer Center (MD Anderson) approved this study (protocol 2017–0591). This study is registered with ClinicalTrials.gov (NCT03367156).

### Study population

Between January 2017 and April 2021, patients were recruited from MD Anderson (Houston, TX, USA) and Lyndon B. Johnson Hospital General Oncology Clinic (LBJ; Houston, TX, USA). Eligibility criteria have been described previously [11, 12]. Briefly, patients were included if they met the following criteria: cancer diagnosis, being 18 years or older, dyspnea rating of 4/10 or greater on the dyspnea NRS in the week before enrollment, Karnofsky performance status [14] of at least 30%, ability to communicate in English or Spanish, and ambulatory patient status at participating centers. Patients were excluded if they had infection requiring antibiotics, delirium at study screening (Memorial Delirium Assessment Scale score > 13/30 [15]), high baseline anxiety score (≥ 15/21 on the Hospital Anxiety and Depression Scale [16]), major postsurgical open wound not healed at time of enrollment, surgery within the past two weeks, uncontrolled diabetes, neutropenia, or anemia, oxygen saturation < 90% despite supplemental oxygen > 6 L per minute (L/min), or preceding chronic systemic corticosteroid use (> 14 days) at time of enrollment. All study participants provided written consent. Patients with a confirmed diagnosis of SARS-CoV-2 infection were also excluded.

### Data collection

We collected demographic data at enrollment, such as age, sex, race, ethnicity and cancer site. Blood specimens were collected venously at enrollment (baseline) and on day 7 (± 2 days) and day 14 (± 2 days) after initial treatment with placebo or dexamethasone. Symptom burden was also assessed at baseline, day 7, and day 14 with the ESAS, which was administered by research staff either in person or via telephone.

### ESAS assessment

The ESAS is a validated questionnaire that measures 10 common symptoms (fatigue, pain, nausea, anxiety, depression, shortness of breath, drowsiness, sleep, appetite, and feeling of well-being) in the past 24 h, each with an 11-point NRS ranging from 0 (no symptom) to 10 (worst possible) [13, 17]. Higher scores indicate worse symptom burden [13, 17]. Dyspnea was the only symptom examined previously [11] and we included it here for completeness. The minimal clinically important difference for ESAS symptoms has been found to be ≤  − 1 point for improvement [18].

### Cytokine measurement

We measured the cytokines tumor necrosis factor (TNF), interleukin-6 (IL-6), interleukin-8 (IL-8), and interleukin-10 (IL-10). IL-10 can be considered either an inflammatory or anti-inflammatory cytokine, dependent on the immunological context [19]. Justification of the use of these cytokines and the exclusion of interleukin-1 beta (IL-1β) measurements from our data was discussed previously [11].

Due to the coronavirus disease 2019 (COVID-19) pandemic, blood collection during the clinical trial was impacted. Restrictions necessitated clinicians switch to telemedicine visits, which limited opportunities for blood draws. Thus, only a portion of the ABCD trial patients provided blood for analysis. Additionally, the supply chain of laboratory reagents was also impacted by COVID-19, resulting in delays in analysis.

We used EDTA vacutainer tubes (K2EDTA, Becton, Dickinson and Company, Franklin Lakes, NJ, USA) to collect specimens. To control for diurnal variations in biomarker levels, collections occurred between 12:00 PM and 4:00 PM [20, 21]. After collection, specimens were transported on ice, centrifuged at 3300 rotations per minute (rpm) for 15 min at 4 °C, aliquoted, and stored at −80 °C for later batch processing.

We quantified plasma cytokine levels with the Quantikine HS ELISA immunoassay (R&D Systems, Minneapolis, MN, USA) in conjunction with the VICTOR5 multilabel plate reader (PerkinElmer, Shelton, CT, USA). We converted optical densities to analyte concentrations with WorkOut data analysis software (PerkinElmer, Shelton, CT, USA) as well as a four-parameter, standardized curve-fitting, logistic regression model. Specimens that had values exceeding the upper range of the standard curve by ≥ 20% were re-run after dilution. This process was used for one IL-8 specimen and 23 IL-6 specimens with a 1:2 or 1:4 dilution, depending on the original value’s magnitude.

To measure assay precision, we computed the coefficient of variation (CV%) as: CV% = (standard deviation [SD] X 100)/mean [22]. We determined a priori that ≤ 15% for intraassay and ≤ 20% for inter-assay CV% were acceptable criteria for precision [23]. We determined intraassay CV% by measuring patient specimens (unknowns) in duplicate [22]. The values for individual plate runs were 3.2–4.0% for TNF, 1.5–4.7% for IL-6, 1.7–9.5% for IL-8, and 6.3–12.8% for IL-10. We determined inter-assay CV% by measuring highly purified controls (R&D Systems, Minneapolis, MN, USA) in duplicate. The average overall CV% between original and repeat assay values was 2.6% for TNF, 1.6% for IL-6, 9.9% for IL-8, and 2.7% for IL-10.

### Statistical analyses

We have described the justification for this study’s sample size previously [12]. To summarize the data distributions, we used descriptive statistics, including means, medians, standard deviations, and interquartile ranges.

We examined differences between patients that provided blood against those who did not by comparing baseline sociodemographic and clinical variables using Mann–Whitney U tests, t-tests for independent groups, and Chi-Square tests as appropriate for the level of data. We transformed cytokine data that did not follow a normal distribution with a logarithmic scale for analysis, and we reported descriptive statistics in picograms per mililiter (pg/mL). Between-group differences between change in symptom intensity from baseline to day 7 and baseline to day 14 were calculated and tested using a standard t-test.

We modeled the change in each symptom’s intensity from baseline to day 7 and baseline to day 14 from baseline cytokine levels using generalized additive models (GAMs) for each symptom. Since there was indication of unstable variability levels over time, the models were kept separate across time. These models fit for change in intensity as a function of time, treatment, and baseline cytokine level. We also incorporated baseline symptom intensity as a covariate. Unless otherwise specified, all variables were fit with linear predictors. The small sample size did not allow for reliable fitting of non-linear relationships. Variables were used without transformation except for IL-8 (raised to power −1/2) and IL-10 (log-transformed). Included variables were baseline symptom intensity level (linear fit), along with baseline cytokine level and treatment and an interaction term for TNF, IL-6, IL-8, and IL-10. P-values for relevant effects were ascertained from the model fit output, including the effect of cytokine on symptom response, broken down by treatment group, and the interaction between those effects to characterize the difference between the slopes.

Further analysis used the results from binomial tests of the signs (positive or negative) of the slope in the placebo and dexamethasone groups at each time point. The null hypothesis posited that the estimated relationship would be approximately equally distributed between positive and negative slopes. Since higher values of ESAS indicate worse symptom burden, a decrease in ESAS values indicated a symptom response to treatment. Thus, a positive slope indicated a negative association, where higher baseline cytokine levels were associated with less benefit over a given time period. Conversely, a negative slope indicated a positive association, where higher baseline cytokine levels were associated with more benefit over a given time period.

The mgcv and lme4 packages in R (version 4.2, R Core Team, Vienna, Austria) were used for data analysis. A *p*-value of 0.05 or less was considered to be statistically significant.

## Results

### Patient characteristics

Patient demographics were described previously [11]. Briefly, of the 128 patients enrolled in the ABCD trial, 45 (35%) provided blood specimens. Thirty-eight (84%) samples were collected at baseline, 24 (53%) were collected on day 7, and 13 (29%) were collected on day 14. Among these 45 patients, 28 (62%) were female, 37 (82%) were Caucasian, and 34 (76%) had respiratory malignancies. No significant differences were found between patients who provided blood and those who did not, except for Hispanic ethnicity (13 [29%] vs. 8 [10%], *p* = 0.005). Eleven (11) patients were randomized to placebo and 34 were randomized to dexamethasone.

### ESAS symptom response to placebo or dexamethasone

Among the 45 patients who provided blood specimens, the placebo group had significantly reduced symptom burden compared to dexamethasone for drowsiness from baseline to day 14 (mean difference placebo-dexamethasone [95% CI] −2.6 [−4.7, −0.5], *p* = 0.02) (Table 1).

**Table 1:** Response of ESAS symptoms to placebo and dexamethasone over time^1,2^

	Placebo		Dexamethasone		Difference (Placebo-Dex)
	*n*	Mean (SD)	*n*	Mean (SD)	Mean (95% CI)
ESAS Pain					
Baseline	11	4.0 (2.8)	34	3.3 (2.3)	0.7 (−1.3, 2.6)
Day 7	9	3.8 (2.3)	28	2.4 (2.2)	1.4 (−0.4, 3.3)
Day 14	9	4.0 (2.6)	28	2.4 (2.5)	1.6 (−0.6, 3.7)
Day 7 – baseline	9	−0.7 (3.5)	28	−1.0 (2.3)	0.7 (−2.4, 3.2)
Day 14 – baseline	9	−0.4 (1.9)	28	−1.0 (2.6)	0.5 (−1.2, 2.2)
ESAS Fatigue					
Baseline	11	5.5 (2.1)	34	5.0 (2.3)	0.6 (−1.0, 2.1)
Day 7	9	5.8 (1.6)	28	4.2 (2.3)	1.6 (0.1, 3.0)
Day 14	9	4.7 (2.7)	28	3.6 (2.1)	1.1 (−1.1, 3.2)
Day 7 – baseline	9	−0.2 (1.4)	28	−0.5 (2.5)	1.0 (−1.1, 1.6)
Day 14 – baseline	9	−1.3 (3.2)	28	−1.1 (2.7)	−0.2 (−2.8, 2.4)
ESAS Nausea					
Baseline	11	0.6 (1.5)	34	1.7 (2.6)	−1.1 (−2.4, 0.2)
Day 7	9	1.7 (2.4)	28	0.79 (1.1)	0.9 (−1.0, 2.7)
Day 14	9	1.2 (1.8)	28	0.64 (1.4)	0.6 (−0.8, 2.0)
Day 7 – baseline	9	0.9 (1.8)	28	−0.8 (2.1)	**2.0 (0.1, 3.1)**
Day 14 – baseline	9	0.4 (1.0)	28	−0.9 (1.9)	**1.3 (0.3, 2.3)**
ESAS Depression					
Baseline	11	2.9 (3.4)	34	1.7 (2.1)	1.2 (−1.2, 3.6)
Day 7	9	2.1 (3.3)	28	1.9 (2.1)	0.2 (−2.4, 2.8)
Day 14	9	1.7 (2.5)	28	1.9 (2.2)	−0.2 (−2.3, 1.9)
Day 7 – baseline	9	−1.4 (2.7)	28	0.3 (2.0)	−1.0 (−3.9, 0.5)
Day 14 – baseline	9	−1.9 (3.4)	28	0.2 (1.9)	−2.1 (−4.8, 0.6)
ESAS Anxiety					
Baseline	11	2.5 (2.9)	34	2.0 (2.0)	0.4 (−1.6, 2.5)
Day 7	9	2.6 (3.1)	28	2.3 (2.2)	0.3 (−2.2, 2.7)
Day 14	9	2.0 (2.4)	28	2.1 (1.6)	−0.1 (−2.0, 1.8)
Day 7 – baseline	9	−0.2 (1.8)	28	0.2 (2.0)	−0.2 (−1.9, 1.1)
Day 14 – baseline	9	−0.8 (2.5)	28	0.0 (2.1)	−0.8 (−2.8, 1.3)
ESAS Drowsiness					
Baseline	11	4.7 (2.5)	34	2.6 (2.7)	2.2 (0.3, 4)
Day 7	9	4.6 (2.2)	28	2.0 (2.1)	2.6 (0.7, 4.4)
Day 14	9	2.9 (2.3)	28	2.3 (2.1)	0.6 (−1.3, 2.5)
Day 7 – baseline	9	−1.2 (2.4)	28	−0.6 (2.1)	0.4 (−2.6, 1.3)
Day 14 – baseline	9	−2.9 (2.4)	28	−0.3 (3)	**−2.6 (−4.7, −0.5)**
ESAS Dyspnea					
Baseline	11	5.5 (1.5)	34	5.3 (1.4)	0.3 (−0.8, 1.4)
Day 7	9	4.0 (2.5)	28	3.7 (1.4)	0.3 (−1.7, 2.3)
Day 14	9	3.8 (2.9)	28	3.1 (1.6)	0.7 (−1.6, 3.0)
Day 7 – baseline	9	−1.9 (3.1)	28	−1.4 (1.5)	0.0 (−2.8, 1.9)
Day 14 – baseline	9	−2.1 (3.3)	28	−2.0 (1.9)	−0.1 (−2.6, 2.5)
ESAS Appetite					
Baseline	11	5.7 (2.8)	34	4.0 (3.1)	1.7 (−0.4, 3.8)
Day 7	9	5.1 (2.6)	28	2.4 (2.1)	2.7 (0.6, 4.8)
Day 14	9	5.0 (2.5)	28	1.5 (2.1)	3.5 (1.4, 5.5)
Day 7 – baseline	9	−1.1 (1.5)	28	−1.9 (3.3)	1.0 (−0.9, 2.4)
Day 14 – baseline	9	−1.2 (2.3)	28	−2.8 (3.1)	1.6 (−0.5, 3.6)
ESAS Sleep					
Baseline	11	5.2 (2.6)	34	4.7 (2.9)	0.5 (−1.5, 2.4)
Day 7	9	3.9 (1.8)	28	3.6 (2.4)	0.3 (−1.3, 1.9)
Day 14	9	3.2 (2.1)	28	3.7 (3.0)	−0.5 (−2.3, 1.4)
Day 7 – baseline	9	−1.4 (3.5)	28	−1.1 (3.1)	−0.2 (−3.2, 2.4)
Day 14 – baseline	9	−2.1 (3.1)	28	−1.0 (3.6)	−1.1 (−3.8, 1.5)
ESAS Well-being					
Baseline	11	4.0 (2.8)	34	4.2 (2.0)	−0.2 (−2.2, 1.8)
Day 7	9	4.2 (3.0)	28	3.5 (2.1)	0.7 (−1.7, 3.1)
Day 14	9	4.4 (2.7)	28	2.7 (2.0)	1.8 (−0.4, 3.9)
Day 7 – baseline	9	−0.3 (1.5)	28	−0.6 (2.1)	0.9 (−1.1, 1.6)
Day 14 – baseline	9	−0.1 (2.1)	28	−1.5 (2.3)	1.4 (−0.4, 3.1)

Abbreviations: *CI* confidence interval, *Dex* dexamethasone, *ESAS* edmonton symptom assessment system,* SD* standard deviation

^1^The ESAS is a validated questionnaire that measures ten common symptoms in the past 4 h (fatigue, pain, nausea, anxiety, depression, shortness of breath, drowsiness, sleep, appetite, and feeling of well-being), each with an 11-point NRS, higher scores indicate worse symptom burden [13, 17]

^2^The between-group difference was calculated and tested using a standard *t*-test. Significant values are bolded

The dexamethasone group had significantly reduced symptom burden compared to placebo for nausea from baseline to day 7 (mean difference placebo-dexamethasone [95% CI] 2.0 [0.1, 3.1], p = 0.03) and baseline to day 14 (1.3 [0.3, 2.3], p = 0.01) (Table 1).

### Patterns of association between symptom response to placebo or dexamethasone and baseline cytokine levels

As described previously, cytokine levels for TNF, IL-6, and IL-8 decreased significantly in the dexamethasone group compared to the placebo group [11]. For IL-10, no significant between-group difference was detected [11].

Using GAMs, we examined the patterns of association between ESAS symptom response to placebo or dexamethasone and baseline cytokine levels (TNF, IL-6, IL-8, and IL-10) from baseline to day 7 and from baseline to day 14. As higher ESAS scores indicate worse symptom burden, symptom response to treatments was indicated by a decrease in ESAS score. In this analysis, a *positive slope*—where higher baseline cytokines were associated with a smaller reduction in ESAS symptom intensity over time—indicated a *negative association* between baseline cytokines and symptom response (since a more negative change in ESAS scores indicates a greater response) (Table 2). Conversely, a *negative slope*—where higher baseline cytokines were associated with greater reduction in ESAS symptom intensity over time—indicated a *positive association* between baseline cytokines and symptom response.

**Table 2:** Associations between baseline cytokine levels and ESAS symptom response to placebo and dexamethasone^a,b^

	Day 7				Day 14			
	Placebo		Dexamethasone		Placebo		Dexamethasone	
	Slope	*p*-value^c^	Slope	*p*-value^c^	Slope	*p*-value^c^	Slope	*p*-value^c^
ESAS Pain								
TNF	3.04	**0.02**	−1.28	0.13	1.48	0.25	−1.35	0.11
IL-6	0.20	0.30	−0.06	0.51	0.16	0.38	−0.02	0.79
IL-8	3.45	0.40	−4.59	0.24	0.29	0.94	−6.44	0.094
IL-10	0.08	0.90	−0.38	0.48	0.76	0.22	0.18	0.71
ESAS Fatigue								
TNF	−0.45	0.66	0.12	0.86	0.34	0.81	0.17	0.86
IL-6	0.03	0.79	0.07	0.32	−0.08	0.61	−0.03	0.76
IL-8	0.49	0.88	−2.01	0.51	−2.51	0.57	−2.76	0.50
IL-10	0.12	0.80	0.38	0.35	1.12	0.084	−0.05	0.93
ESAS Nausea								
TNF	1.75	**0.031**	0.25	0.64	1.18	**0.034**	0.05	0.89
IL-6	0.07	0.48	0.05	0.43	0.08	0.22	0.01	0.88
IL-8	5.48	**0.025**	−0.89	0.71	2.56	0.13	−1.68	0.32
IL-10	0.42	0.30	−0.09	0.78	0.18	0.53	−0.06	0.80
ESAS Depression								
TNF	−0.03	0.98	−0.53	0.57	−1.36	0.30	−1.25	0.17
IL-6	0.08	0.68	0.05	0.58	−0.05	0.80	−0.08	0.42
IL-8	4.61	0.21	0.55	0.88	−1.28	0.72	−5.82	0.12
IL-10	1.25	0.051	−0.06	0.90	0.84	0.22	0.26	0.61
ESAS Anxiety								
TNF	1.21	0.32	−0.43	0.61	−0.63	0.53	−1.01	0.16
IL-6	−0.02	0.88	−0.0004	1.00	−0.16	0.18	−0.13	0.062
IL-8	3.86	0.30	−0.18	0.96	−4.67	0.11	−4.14	0.15
IL-10	0.37	0.50	−0.30	0.51	0.33	0.48	−0.20	0.61
ESAS Drowsiness								
TNF	2.00	0.06	−0.54	0.46	0.86	0.53	0.42	0.67
IL-6	0.25	0.053	−0.01	0.87	0.27	0.10	0.01	0.90
IL-8	3.88	0.24	−1.49	0.65	6.45	0.11	0.69	0.86
IL-10	1.15	**0.017**	0.05	0.88	0.97	0.12	−0.32	0.51
ESAS Dyspnea								
TNF	2.36	**0.019**	−0.66	0.33	3.37	**0.0041**	−0.46	0.55
IL-6	0.51	** < 0.0001**	0.0034	0.94	0.61	** < 0.0001**	0.01	0.87
IL-8	7.07	**0.014**	−3.20	0.24	7.62	**0.024**	−4.10	0.21
IL-10	1.21	**0.020**	−0.44	0.23	1.07	**0.094**	−0.34	0.47
ESAS Appetite								
TNF	2.40	0.063	0.17	0.84	1.83	0.16	0.38	0.67
IL-6	0.40	**0.013**	0.05	0.54	0.18	0.29	−0.05	0.60
IL-8	6.25	0.10	−3.18	0.40	8.53	**0.021**	−3.33	0.35
IL-10	1.13	0.065	−0.0008	1.00	1.47	**0.021**	−0.01	0.97
ESAS Sleep								
TNF	−1.62	0.23	−0.91	0.35	−1.16	0.47	−0.14	0.90
IL-6	−0.12	0.48	0.02	0.86	−0.07	0.73	0.05	0.64
IL-8	−4.58	0.27	−3.76	0.37	−4.25	0.38	−3.92	0.42
IL-10	−0.42	0.49	−0.61	0.22	0.99	0.19	−0.17	0.78
ESAS Well-being								
TNF	1.60	0.14	0.02	0.98	0.48	0.70	−0.74	0.40
IL-6	0.12	0.37	0.07	0.37	0.26	0.073	−0.16	0.056
IL-8	4.27	0.23	−2.76	0.40	4.07	0.29	−7.04	0.056
IL-10	0.69	0.25	−0.22	0.60	0.91	0.19	0.04	0.93

Abbreviations: *ESAS* edmonton symptom assessment system, *IL* interleukin, *TNF* tumor necrosis factor

^a^The ESAS is a validated questionnaire that measures ten common symptoms in the past 4 h (fatigue, pain, nausea, anxiety, depression, shortness of breath, drowsiness, sleep, appetite, and feeling of well-being), each with an 11-point NRS, higher scores indicate worse symptom burden [13, 17]

^b^Cytokines were measured venously by Quantikine HS ELISA immunoassay

^c^P-values for relevant effects were ascertained at each time from the additive model fit, including the effect of cytokine on symptom response, broken down by treatment group, and the interaction between those effects to characterize the difference between the slopes

For placebo response from baseline to day 7, we observed a significant negative association between ≥ 1 of the measured cytokines’ baseline levels and multiple symptoms, including pain, nausea, drowsiness, dyspnea, and appetite (*p* < 0.05 for all symptoms) (Table 2), suggesting that patients with higher baseline cytokine levels were less likely to report a reduction in symptoms from placebo. We also observed a similar relationship for depression, anxiety, and well-being, albeit not statistically significant (Table 2). We found no consistent pattern in placebo response for fatigue and sleep (Table 2).

For dexamethasone response from baseline to day 7, we observed a positive association between ≥ 1 of the measured cytokines’ baseline levels and multiple symptoms, including pain, fatigue, drowsiness, dyspnea, appetite, sleep, and well-being. However, these were not statistically significant (Table 2). We found no consistent pattern in dexamethasone response for nausea, depression, or anxiety (Table 2).

For placebo response from baseline to day 14, we observed a significant negative association between ≥ 1 of the measured cytokines’ baseline levels and nausea, dyspnea, and appetite (*p* < 0.05 for all symptoms) (Table 2). We also observed a similar relationship for pain, fatigue, drowsiness, and well-being, albeit not statistically significant (Table 2). We found no consistent pattern in placebo response for depression, anxiety, or sleep (Table 2).

For dexamethasone response from baseline to day 14, we observed a positive association between ≥ 1 of the measured cytokines’ baseline levels and multiple symptoms, similar to what was observed for day 7. However, the associated symptoms were slightly different: pain, fatigue, nausea, depression, anxiety, dyspnea, appetite, sleep, and well-being. None of these associations were statistically significant (Table 2). We found no consistent pattern in dexamethasone response for drowsiness (Table 2).

### Patterns of association in exploratory analysis

We also performed binomial tests on the sign of the slope for both placebo and dexamethasone groups. The null hypothesis for this analysis was that the estimated relationship would be approximately equally distributed between positive and negative slopes.

For the placebo group, we observed a positive slope (negative association) between baseline cytokine levels and symptom response for 33 of 40 combinations for day 7 (*p* < 0.001) and 29 of 40 combinations for day 14 (p = 0.006) (Table 3), further suggesting that patients with higher baseline cytokine levels were less likely to report a reduction in symptoms from placebo.

**Table 3:** Trends in exploratory analysis^a^

	Placebo^**b**^				Dexamethasone^b^			
	Day 7		Day 14		Day 7		Day 14	
	Parameters	*p*-value	Parameters	*p*-value	Parameters	*p-*value	Parameters	*p*-value
TNF	7/10	0.34	7/10	0.34	4/10	0.75	4/10	0.75
IL-6	8/10	0.11	6/10	0.75	7/10	0.34	4/10	0.75
IL-8	9/10	0.02	6/10	0.75	1/10	0.02	1/10	0.02
IL-10	9/10	0.02	10/10	0.002	2/10	0.11	3/10	0.34
Total	33/40	< 0.001	29/40	0.006	14/40	0.08	12/40	0.02

^a^Binomial tests of the signs (positive or negative) of the slope in the placebo and dexamethasone groups at each time point were further analyzed; the null hypothesis posited that the estimated relationship would be approximately equally distributed between positive and negative slopes

^b^The rate of positive slopes (negative associations) was assessed marginally and by cytokine against the null hypothesis of no association across the 40 outcomes, 10 per cytokine

For the dexamethasone group, we observed a positive slope (negative association) between baseline cytokine levels and symptom response for 14 of 40 combinations for day 7 (*p *= 0.08) and 12 of 40 combinations for day 14 (*p* = 0.02) (Table 3).

## Discussion

In this exploratory analysis of a randomized clinical trial of patients with cancer, we found that higher levels of baseline cytokines were associated with a significantly smaller treatment effect for the placebo group. This pattern of association was observed across a diverse set of symptoms, highlighting the role of inflammation in the placebo response, with potential implications for future clinical trial design. These findings are considered preliminary and have yet to be confirmed in larger studies.

Many clinical trials involving patient-reported outcomes have observed a large placebo response, contributing to a muted difference between placebo and intervention groups and ultimately resulting in negative findings [12, 24–29]. We observed this in our previous placebo-controlled trial examining the use of dexamethasone for dyspnea in patients with cancer, as well as in other cancer trials using dexamethasone for fatigue and anorexia [12, 24, 25]. The mechanisms of the placebo response are not well understood, although research indicates that expectation of an effect, preconceived bias, self-efficacy, co-interventions, and interaction with study staff may increase the incidence of placebo response [30, 31]. The ability to distinguish patients who are more or less likely to have a placebo response is of scientific interest for several reasons—both to better understand the mechanism of placebo response and to select an appropriate population for interventional trials.

To date, only a handful of placebo-controlled trials have identified inflammatory biomarkers as potential predictors of placebo response. Several trials, largely in depression, found that placebo responders were more likely to have lower levels of baseline inflammation [5–7, 9]. We recently reported that higher cytokine levels were associated with a lower dyspnea response in the placebo group [11], prompting us to conduct the current study to investigate if this relationship applies to other symptoms (and thus placebo response more globally). To our knowledge, our analysis is the first to examine the patterns of association between placebo response for an array of symptoms and baseline inflammatory biomarker levels. In doing so, we found a negative association between baseline inflammation and placebo response. We postulate that inflammation may modulate the placebo response in the context of symptom expression. The inflammatory response has been found to modulate dyspnea pathways in part by upregulating/sensitizing the limbic system, somatosensory cortex, and thalamocortical pathways [32–37]. Interestingly, placebo as an intervention has also been found to dampen signals in the limbic system and somatosensory cortex, which are regions of the brain also targeted by the inflammatory response [38–42]. This interaction may have mechanistic implications for how inflammation could potentially attenuate the placebo response.

This pattern is of particular interest for the post hoc analysis presented here, as patients were not enrolled specifically for these secondary symptoms. The average intensity of these symptoms at baseline was generally low, making it less likely that we would detect a response. Furthermore, compared to the primary symptom of dyspnea, patients may have been less likely to think that the study treatment would improve their secondary symptoms, minimizing any pre-conceived expectations that might have further precipitated the placebo response. Thus, it is notable that we detected a significant association between baseline cytokine levels and placebo response across multiple symptoms. Taken together with limited existing literature, our data suggest that this negative association between placebo response and baseline levels of inflammatory markers may be agnostic to symptoms. This pattern of association raises the possibility that a central mechanism, which affects the experience/perception of multiple symptoms, may be impacted by baseline cytokine levels. Notably, the midbrain and its cortical projections are involved in the regulation of multiple symptoms, including pain, depression, and anxiety [43–45], and multiple functions within the midbrain, such as dopamine and serotonin regulation, have been found to be affected by pro-inflammatory cytokines [46, 47]. However, we cannot exclude the possibility that the responses across multiple symptoms were associated with dyspnea response in the placebo group.

It is also notable that not every symptom in our dataset had a significant association with baseline cytokine levels for placebo response, and even symptoms that did have a significant association were not always associated with all cytokines across every time period. This finding may be explained partly by the small sample size and high variability in cytokine levels and symptom expression. Since the baseline intensity of symptoms was very low, a floor effect may have also limited our findings. Our exploratory analysis examining recurrent patterns in the relationship between baseline cytokines and symptom response further supports the overall hypothesis of a negative association between baseline cytokine levels and symptom response in the placebo group. Further research is needed to confirm these findings.

Our findings have important potential implications for research and clinical practice. From the research perspective, identifying biomarkers of placebo response may facilitate targeted recruitment of patients most likely to respond to treatment in clinical trials, effectively increasing the effect size, allowing for clearer interpretation, and reducing trial size and cost. Furthermore, these findings hint at the potential mechanistic associations between inflammation, placebo response, and symptom perception, inviting further investigation on this topic. From the clinical perspective, clinicians may be able to provide relief for symptoms that are difficult to manage with current treatments by leveraging placebo response in select patients identified by biomarkers [48, 49].

This study had several limitations. First, this trial was terminated earlier than expected for futility, which restricted the number of patients in the secondary analysis. Second, this study’s randomization scheme was 2:1 dexamethasone:placebo. Thus, there were a smaller number of patients randomized to placebo for this analysis. Third, as this study occurred during the initial COVID-19 pandemic, only a small subgroup of patients provided blood work. However, we detected no significant differences between patients who provided blood and those who did not, except for Hispanic ethnicity. Fourth, we examined the effect of multiple cytokines and multiple symptoms, which could lead to false positives through multiple testing. However, the effects were observed across multiple symptoms, suggesting an underlying mechanism. It is worth noting that for some cytokines, particularly IL-10, the overlap between the distributions was small, indicating that the interaction effects were observed across a small subset of the cytokine values. Fifth, placebo response may have been attributed to non-specific effects, such as regression to the mean, expectation effects, or Hawthorne effect. Sixth, although this is a randomized trial and the co-interventions are expected to be mostly balanced, we cannot exclude the possibility that any imbalances may have impacted the symptom intensity in each study group. Further research is needed to understand the impact of these factors. Seventh, this was a post hoc, exploratory analysis based on available data. These findings should be considered for hypothesis-generating purposes only.

## Conclusions

Our study found patterns of negative association between baseline cytokine levels and placebo response for multiple symptoms in patients with cancer, and our findings highlight the potential mechanistic link between baseline inflammation and a central mechanism for placebo response. More research is needed to further explore the mechanisms of placebo response.

## Data Availability

Individual participant data that underlie the results reported in this article, after deidentification, are available immediately and ending five years after publication to investigators whose proposed use of the data has been approved by the Institutional Review Board at the University of Texas MD Anderson Cancer Center. Proposals should be directed to dhui@mdanderson.org. To gain access, data requestors will need to sign a data access agreement.

## References

[1] Roji R, Stone P, Ricciardi F, Candy B (2020) Placebo response in trials of drug treatments for cancer-related fatigue: a systematic review, meta-analysis and meta-regression. BMJ Support Palliat Care 10:385–394. 10.1136/bmjspcare-2019-002163 32046962 10.1136/bmjspcare-2019-002163 PMC7691807

[2] Feliciano JL, Waldfogel JM, Sharma R, Zhang A, Gupta A, Sedhom R, Day J, Bass EB, Dy SM (2021) Pharmacologic interventions for breathlessness in patients with advanced cancer: a systematic review and meta-analysis. JAMA Netw Open 4:e2037632. 10.1001/jamanetworkopen.2020.37632 33630086 10.1001/jamanetworkopen.2020.37632 PMC7907959

[3] Hardy J, Quinn S, Fazekas B, Plummer J, Eckermann S, Agar M, Spruyt O, Rowett D, Currow DC (2012) Randomized, double-blind, placebo-controlled study to assess the efficacy and toxicity of subcutaneous ketamine in the management of cancer pain. J Clin Oncol 30:3611–3617. 10.1200/JCO.2012.42.1081 22965960 10.1200/JCO.2012.42.1081

[4] Hunter CN, Abdel-Aal HH, Elsherief WA, Farag DE, Riad NM, Alsirafy SA (2021) Mirtazapine in cancer-associated anorexia and cachexia: a double-blind placebo-controlled randomized trial. J Pain Symptom Manage 62:1207–1215. 10.1016/j.jpainsymman.2021.05.017 34051293 10.1016/j.jpainsymman.2021.05.017

[5] Raison CL, Rutherford RE, Woolwine BJ, Shuo C, Schettler P, Drake DF, Haroon E, Miller AH (2013) A randomized controlled trial of the tumor necrosis factor antagonist infliximab for treatment-resistant depression: the role of baseline inflammatory biomarkers. JAMA Psychiat 70:31–41. 10.1001/2013.jamapsychiatry.4 10.1001/2013.jamapsychiatry.4 PMC4015348 22945416

[6] Simon MS, Burger B, Weidinger E, Arteaga-Henriquez G, Zill P, Musil R, Drexhage HA, Muller N (2021) Efficacy of sertraline plus placebo or add-on celecoxib in major depressive disorder: macrophage migration inhibitory factor as a promising biomarker for remission after sertraline-results from a randomized controlled clinical trial. Front Psychiatry 12:615261. 10.3389/fpsyt.2021.615261 34646168 10.3389/fpsyt.2021.615261 PMC8504576

[7] Raison CL, Siu C, Pikalov A, Tocco M, Loebel A (2020) C-reactive protein and response to lurasidone treatment in children and adolescents with bipolar I depression: results from a placebo-controlled trial. Brain Behav Immun 84:269–274. 10.1016/j.bbi.2019.12.010 31857217 10.1016/j.bbi.2019.12.010

[8] Kokkotou E, Conboy LA, Ziogas DC, Quilty MT, Kelley JM, Davis RB, Lembo AJ, Kaptchuk TJ (2010) Serum correlates of the placebo effect in irritable bowel syndrome. Neurogastroenterol Motil 22:285-e281. 10.1111/j.1365-2982.2009.01440.x 20028464 10.1111/j.1365-2982.2009.01440.x PMC2852478

[9] Raison CL, Pikalov A, Siu C, Tsai J, Koblan K, Loebel A (2018) C-reactive protein and response to lurasidone in patients with bipolar depression. Brain Behav Immun 73:717–724. 10.1016/j.bbi.2018.08.009 30102967 10.1016/j.bbi.2018.08.009

[10] Torres A, Sibila O, Ferrer M, Polverino E, Menendez R, Mensa J, Gabarrus A, Sellares J, Restrepo MI, Anzueto A, Niederman MS, Agusti C (2015) Effect of corticosteroids on treatment failure among hospitalized patients with severe community-acquired pneumonia and high inflammatory response: a randomized clinical trial. JAMA 313:677–686. 10.1001/jama.2015.88 25688779 10.1001/jama.2015.88

[11] Hui D, Hanneman SK, Jennings K, Ontai A, Cron S, Bruera E (2024) Predictive biomarkers of dyspnea response to dexamethasone and placebo in cancer patients. J Pain Symptom Manage. 10.1016/j.jpainsymman.2024.07.003 39297834 10.1016/j.jpainsymman.2024.07.003

[12] Hui D, Puac V, Shelal Z, Dev R, Hanneman SK, Jennings K, Ma H, Urbauer DL, Shete S, Fossella F, Liao Z, Blumenschein G Jr., Chang JY, O’Reilly M, Gandhi SJ, Tsao A, Mahler DA, Bruera E (2022) Effect of dexamethasone on dyspnoea in patients with cancer (ABCD): a parallel-group, double-blind, randomised, controlled trial. Lancet Oncol 23:1321–1331. 10.1016/S1470-2045(22)00508-3 10.1016/S1470-2045(22)00508-3 PMC10618956 36087590

[13] Hui D, Bruera E (2017) The Edmonton symptom assessment system 25 years later: past, present, and future developments. J Pain Symptom Manage 53:630–643. 10.1016/j.jpainsymman.2016.10.370 28042071 10.1016/j.jpainsymman.2016.10.370 PMC5337174

[14] Mor V, Laliberte L, Morris JN, Wiemann M (1984) The Karnofsky Performance Status Scale. An examination of its reliability and validity in a research setting. Cancer 53:2002–2007. 10.1002/1097-0142(19840501)53:9&lt;2002::aid-cncr2820530933&gt;3.0.co;2-w 6704925 10.1002/1097-0142(19840501)53:9<2002::aid-cncr2820530933>3.0.co;2-w

[15] Breitbart W, Rosenfeld B, Roth A, Smith MJ, Cohen K, Passik S (1997) The Memorial Delirium Assessment Scale. J Pain Symptom Manage 13:128–137. 10.1016/s0885-3924(96)00316-8 9114631 10.1016/s0885-3924(96)00316-8

[16] Bjelland I, Dahl AA, Haug TT, Neckelmann D (2002) The validity of the hospital anxiety and depression scale. An updated literature review. J Psychosom Res 52:69–77. 10.1016/s0022-3999(01)00296-3 11832252 10.1016/s0022-3999(01)00296-3

[17] Bruera E, Kuehn N, Miller MJ, Selmser P, Macmillan K (1991) The Edmonton symptom assessment system (ESAS): a simple method for the assessment of palliative care patients. J Palliat Care 7:6–9 1714502

[18] Hui D, Shamieh O, Paiva CE, Perez-Cruz PE, Kwon JH, Muckaden MA, Park M, Yennu S, Kang JH, Bruera E (2015) Minimal clinically important differences in the Edmonton symptom assessment scale in cancer patients: a prospective, multicenter study. Cancer 121:3027–3035. 10.1002/cncr.29437 26059846 10.1002/cncr.29437 PMC4595042

[19] Carlini V, Noonan DM, Abdalalem E, Goletti D, Sansone C, Calabrone L, Albini A (2023) The multifaceted nature of IL-10: regulation, role in immunological homeostasis and its relevance to cancer, COVID-19 and post-COVID conditions. Front Immunol 14:1161067. 10.3389/fimmu.2023.1161067 37359549 10.3389/fimmu.2023.1161067 PMC10287165

[20] Petrovsky N, McNair P, Harrison LC (1998) Diurnal rhythms of pro-inflammatory cytokines: regulation by plasma cortisol and therapeutic implications. Cytokine 10:307–312. 10.1006/cyto.1997.0289 9617577 10.1006/cyto.1997.0289

[21] Knudsen LS, Christensen IJ, Lottenburger T, Svendsen MN, Nielsen HJ, Nielsen L, Horslev-Petersen K, Jensen JE, Kollerup G, Johansen JS (2008) Pre-analytical and biological variability in circulating interleukin 6 in healthy subjects and patients with rheumatoid arthritis. Biomarkers 13:59–78. 10.1080/13547500701615017 17852075 10.1080/13547500701615017

[22] Hanneman SK, Cox CD, Green KE, Kang DH (2011) Estimating intra- and inter-assay variability in salivary cortisol. Biol Res Nurs 13:243–250. 10.1177/1099800411404061 21498487 10.1177/1099800411404061

[23] Chaturvedi AK, Kemp TJ, Pfeiffer RM, Biancotto A, Williams M, Munuo S, Purdue MP, Hsing AW, Pinto L, McCoy JP, Hildesheim A (2011) Evaluation of multiplexed cytokine and inflammation marker measurements: a methodologic study. Cancer Epidemiol Biomarkers Prev 20:1902–1911. 10.1158/1055-9965.EPI-11-0221 21715603 10.1158/1055-9965.EPI-11-0221 PMC3400264

[24] Yennurajalingam S, Frisbee-Hume S, Palmer JL, Delgado-Guay MO, Bull J, Phan AT, Tannir NM, Litton JK, Reddy A, Hui D, Dalal S, Massie L, Reddy SK, Bruera E (2013) Reduction of cancer-related fatigue with dexamethasone: a double-blind, randomized, placebo-controlled trial in patients with advanced cancer. J Clin Oncol 31:3076–3082. 10.1200/JCO.2012.44.4661 23897970 10.1200/JCO.2012.44.4661

[25] Currow DC, Glare P, Louw S, Martin P, Clark K, Fazekas B, Agar MR (2021) A randomised, double blind, placebo-controlled trial of megestrol acetate or dexamethasone in treating symptomatic anorexia in people with advanced cancer. Sci Rep 11:2421. 10.1038/s41598-021-82120-8 33510313 10.1038/s41598-021-82120-8 PMC7844230

[26] Fournier JC, DeRubeis RJ, Hollon SD, Dimidjian S, Amsterdam JD, Shelton RC, Fawcett J (2010) Antidepressant drug effects and depression severity: a patient-level meta-analysis. JAMA 303:47–53. 10.1001/jama.2009.1943 20051569 10.1001/jama.2009.1943 PMC3712503

[27] Finniss DG, Kaptchuk TJ, Miller F, Benedetti F (2010) Biological, clinical, and ethical advances of placebo effects. Lancet 375:686–695. 10.1016/s0140-6736(09)61706-2 20171404 10.1016/S0140-6736(09)61706-2 PMC2832199

[28] Kirsch I, Deacon BJ, Huedo-Medina TB, Scoboria A, Moore TJ, Johnson BT (2008) Initial severity and antidepressant benefits: a meta-analysis of data submitted to the Food and Drug Administration. PLoS Med 5:e45. 10.1371/journal.pmed.0050045 18303940 10.1371/journal.pmed.0050045 PMC2253608

[29] Hui D, Pacheco S, Nguyen L, Jennings K, de la Cruz V, Stanton P, Laureano R, Ontai A, Mahler D, Bruera E (2025) Efficacy of fentanyl buccal tablet and morphine for exertional dyspnoea in patients with cancer: a double-blind, placebo-controlled, randomised clinical trial. Thorax. 10.1136/thorax-2025-222970 10.1136/thorax-2025-222970 41115797

[30] Horing B, Weimer K, Muth ER, Enck P (2014) Prediction of placebo responses: a systematic review of the literature. Front Psychol 5:1079. 10.3389/fpsyg.2014.01079 25324797 10.3389/fpsyg.2014.01079 PMC4181242

[31] Petrie KJ, Rief W (2019) Psychobiological mechanisms of placebo and nocebo effects: pathways to improve treatments and reduce side effects. Annu Rev Psychol 70:599–625. 10.1146/annurev-psych-010418-102907 30110575 10.1146/annurev-psych-010418-102907

[32] Labrenz F, Wrede K, Forsting M, Engler H, Schedlowski M, Elsenbruch S, Benson S (2016) Alterations in functional connectivity of resting state networks during experimental endotoxemia - an exploratory study in healthy men. Brain Behav Immun 54:17–26. 10.1016/j.bbi.2015.11.010 26597151 10.1016/j.bbi.2015.11.010

[33] Benson S, Rebernik L, Wegner A, Kleine-Borgmann J, Engler H, Schlamann M, Forsting M, Schedlowski M, Elsenbruch S (2015) Neural circuitry mediating inflammation-induced central pain amplification in human experimental endotoxemia. Brain Behav Immun 48:222–231. 10.1016/j.bbi.2015.03.017 25882910 10.1016/j.bbi.2015.03.017

[34] Qin H, Duan G, Zhang Q, Lai Z, Chen Y, Lai Y, Wu Y, Liu Z, Zhou K, Zhang Y, Lin S, Sun R, Li S, Ou Y, Wu R, Chen Z, Liang L, Deng D (2025) Alterations of white matter connectivity in thalamic-frontal pathways associated with inflammation in premenstrual syndrome. Psychoradiology 5:kkaf027. 10.1093/psyrad/kkaf027 41200094 10.1093/psyrad/kkaf027 PMC12586991

[35] Scheuren PS, Calvo M (2024) Exploring neuroinflammation: a key driver in neuropathic pain disorders. Int Rev Neurobiol 179:311–338. 10.1016/bs.irn.2024.10.009 39580216 10.1016/bs.irn.2024.10.009

[36] Kullmann JS, Grigoleit JS, Lichte P, Kobbe P, Rosenberger C, Banner C, Wolf OT, Engler H, Oberbeck R, Elsenbruch S, Bingel U, Forsting M, Gizewski ER, Schedlowski M (2013) Neural response to emotional stimuli during experimental human endotoxemia. Hum Brain Mapp 34:2217–2227. 10.1002/hbm.22063 22461242 10.1002/hbm.22063 PMC6870425

[37] Kelleher EM, Meouchi R, Irani A (2025) Beyond inflammation: why understanding the brain matters in inflammatory arthritis. Arthritis Care Res (Hoboken). 10.1002/acr.25694 41236146 10.1002/acr.25694 PMC12826092

[38] Elsenbruch S, Kotsis V, Benson S, Rosenberger C, Reidick D, Schedlowski M, Bingel U, Theysohn N, Forsting M, Gizewski ER (2012) Neural mechanisms mediating the effects of expectation in visceral placebo analgesia: an fMRI study in healthy placebo responders and nonresponders. Pain 153:382–390. 10.1016/j.pain.2011.10.036 22136749 10.1016/j.pain.2011.10.036

[39] Price DD, Craggs J, Verne GN, Perlstein WM, Robinson ME (2007) Placebo analgesia is accompanied by large reductions in pain-related brain activity in irritable bowel syndrome patients. Pain 127:63–72. 10.1016/j.pain.2006.08.001 16963184 10.1016/j.pain.2006.08.001

[40] Ellingsen DM, Wessberg J, Eikemo M, Liljencrantz J, Endestad T, Olausson H, Leknes S (2013) Placebo improves pleasure and pain through opposite modulation of sensory processing. Proc Natl Acad Sci U S A 110:17993–17998. 10.1073/pnas.1305050110 24127578 10.1073/pnas.1305050110 PMC3816412

[41] Wager TD, Atlas LY (2015) The neuroscience of placebo effects: connecting context, learning and health. Nat Rev Neurosci 16:403–418. 10.1038/nrn3976 26087681 10.1038/nrn3976 PMC6013051

[42] Huneke NTM, Aslan IH, Fagan H, Phillips N, Tanna R, Cortese S, Garner M, Baldwin DS (2022) Functional neuroimaging correlates of placebo response in patients with depressive or anxiety disorders: a systematic review. Int J Neuropsychopharmacol 25:433–447. 10.1093/ijnp/pyac009 35078210 10.1093/ijnp/pyac009 PMC9211006

[43] McLaughlin I, Dani JA, De Biasi M (2017) The medial habenula and interpeduncular nucleus circuitry is critical in addiction, anxiety, and mood regulation. J Neurochem 142(2):130–143. 10.1111/jnc.14008 28791703 10.1111/jnc.14008 PMC6740332

[44] Zhang H, Li L, Zhang X, Ru G, Zang W (2024) Role of the dorsal raphe nucleus in pain processing. Brain Sci. 10.3390/brainsci14100982 39451996 10.3390/brainsci14100982 PMC11506261

[45] Zhang H, Zhu Z, Ma WX, Kong LX, Yuan PC, Bu LF, Han J, Huang ZL, Wang YQ (2024) The contribution of periaqueductal gray in the regulation of physiological and pathological behaviors. Front Neurosci 18:1380171. 10.3389/fnins.2024.1380171 38650618 10.3389/fnins.2024.1380171 PMC11034386

[46] Litteljohn D, Mangano E, Clarke M, Bobyn J, Moloney K, Hayley S (2010) Inflammatory mechanisms of neurodegeneration in toxin-based models of Parkinson’s disease. Parkinsons Dis 2011:713517. 10.4061/2011/713517 21234362 10.4061/2011/713517 PMC3018622

[47] Miller AH, Haroon E, Raison CL, Felger JC (2013) Cytokine targets in the brain: impact on neurotransmitters and neurocircuits. Depress Anxiety 30:297–306. 10.1002/da.22084 23468190 10.1002/da.22084 PMC4141874

[48] Hoenemeyer TW, Baidwan NK, Hall K, Kaptchuk TJ, Fontaine KR, Mehta TS (2021) An exploratory analysis of the association between catechol-O-methyltransferase and response to a randomized open-label placebo treatment for cancer-related fatigue. Front Psychiatry 12:684556. 10.3389/fpsyt.2021.684556 34267689 10.3389/fpsyt.2021.684556 PMC8275998

[49] Carvalho C, Caetano JM, Cunha L, Rebouta P, Kaptchuk TJ, Kirsch I (2016) Open-label placebo treatment in chronic low back pain: a randomized controlled trial. Pain 157:2766–2772. 10.1097/j.pain.0000000000000700 27755279 10.1097/j.pain.0000000000000700 PMC5113234

